# Estimating the Similarity between Protein Pockets

**DOI:** 10.3390/ijms232012462

**Published:** 2022-10-18

**Authors:** Merveille Eguida, Didier Rognan

**Affiliations:** Laboratoire d’Innovation Thérapeutique, UMR7200 CNRS-Université de Strasbourg, 67400 Illkirch, France

**Keywords:** binding site, alignment, off-target, library design, virtual screening, machine learning, artificial intelligence drug design

## Abstract

With the exponential increase in publicly available protein structures, the comparison of protein binding sites naturally emerged as a scientific topic to explain observations or generate hypotheses for ligand design, notably to predict ligand selectivity for on- and off-targets, explain polypharmacology, and design target-focused libraries. The current review summarizes the state-of-the-art computational methods applied to pocket detection and comparison as well as structural druggability estimates. The major strengths and weaknesses of current pocket descriptors, alignment methods, and similarity search algorithms are presented. Lastly, an exhaustive survey of both retrospective and prospective applications in diverse medicinal chemistry scenarios illustrates the capability of the existing methods and the hurdle that still needs to be overcome for more accurate predictions.

## 1. Introduction

In living organisms, biological processes are regulated through specific molecular recognition at local surfaces. Proteins, one of the major biomolecules composing our cells, interact with different partners: other proteins, peptides, nucleic acids, small molecules, and transition metals. The exploration of the proteome, considering amino acid sequences, makes it possible to rapidly compare proteins but does not necessarily indicate whether potential cavities at their surfaces will be conserved or not. Hence, structure conservation does not always mirror sequence homology [1]. Progress in molecular and structural biology have enabled us to uncover the three-dimensional (3D) structure of proteins, either by X-ray diffraction [2], nuclear magnetic resonance (NMR) [3], or more recently cryo-electron microscopy (cryo-EM) at an atomic scale [4], all approaches being now integrated [5]. Characterizing the binding cavities for small molecules has bolstered the rise of structure-based drug design [6]. Supported by the outlooks and successful case studies, many methods have been developed in the last three decades. The bottleneck of protein cavity comparison is common to all similarity estimates—similarity is a non-measurable characteristic that depends on the considered aspects. Instead, derived hypotheses (e.g., function, ligand binding) are further evaluated. This presents many challenges for benchmarking methods and highlights the importance of carefully designing datasets in retrospective studies. For users as well as developers, knowing where we come from and what has been achieved in the field enables realistic expectations and spot limitations to be addressed by future developments.

Structure-based algorithms for protein site comparison emerged in the 1970s, a decade marked by the establishment of the Protein Data Bank (PDB) [7] and the deposit of a few structures. Initially, efforts were made to compare protein 3D structural motifs independently of sequence order and gaps. Computer vision approaches [8] were applied in structural biology for similar substructure identification even in the absence of sequence homology via rigid body alignments. Protein functions could be predicted from a database of known 3D templates, by querying or inferring protein active sites [9]. Beyond the functional annotations, cavity alignment and comparison rapidly became promising for the rational design of proteins and ligands, since similar 3D arrangements of surface motifs may be involved in similar molecular recognition events [10].

The path from the earlier to the current site comparison methods involved several implementations. It was common for the user to define researched features (e.g., set of atom/residues distances defining a motif: catalytic triads, similar ligands) from prior knowledge to initiate the search [11]. The subsequent advantages are a better control of the comparison, an easier selection of relevant matches, and the reliability of the solutions. Progressively, methods allowing for the automatic identification of pockets [12,13,14,15,16] and of relevant matched patterns opened the doors to the analysis of the relationships between evolutionally and structurally remote members of an entire database, without any a priori judgment [17,18,19,20,21]. Such predictions led to unexpected findings with implications for drug design [22,23]. Screening large databases requires effective computing time. Together with the progress of computing technologies, fast methods were introduced, but often at the cost of interpretability [24,25,26].

The repertoire of possible comparison algorithms is tailored to the representation made of the pocket [27]. Pocket representation is a way to provide structured information to the algorithm for exploration. Once delimited in the protein, a pocket can be modeled as a list of amino acids, graphs, or unconnected pseudo atoms, among other possibilities. The geometrical constraints of alpha carbon tuples were extensively used to identify equivalenced areas [28,29,30]. Other cavity descriptors further encode the chemical properties of atoms or residues, hence reducing redundancy in the possible matches [17,31,32]. The intricacy of the representation lays in finding a good balance between fuzziness (with a risk of false positive matches) and exhaustiveness (with a risk of missing remote similarities). In any case, similarity can only be properly reported with a fair scoring function. The scoring scheme aims at quantifying how two pockets resemble or differ. Often, a score threshold is applied in screening campaigns for decision making. How to assign the value of that threshold and assess the significance of that similarity is a genuine question raised by earlier studies [24,33,34].

In practice, the variability of the pocketome [35] in terms of size, solvent accessibility, and flexibility constitutes an obstacle to the performance of binding site comparison methods, as it is for other structure-based approaches [36]. It is perceived that comparing subpockets instead of entire cavities might better handle the conformational variations, typically induced by ligand binding. Noteworthy, the ability to detect local and global similarities is suitable for different purposes. As the reader will notice, different parameters entail the success of protein cavity comparisons, as discussed by previous articles [23,37,38,39]. In this review, we will provide a most recent and broad overview of all stages involved in pocket comparison, from the prediction of ligand binding sites to the evaluation and prospective applications in drug design.

## 2. Pocket Detection and Druggability Estimate

The identification of potential interaction sites is crucial to structure-based approaches and constitutes the very first step of binding site comparison. Contact surfaces exhibit different geometric and physicochemical characteristics according to the nature of the binding partner (proteins, peptides, nucleic acids, small molecules, and transition metals). For example, small molecule interaction sites are buried clefts, while protein–protein interaction interfaces are rather flat and hydrophobic [40]. Although the available methods for binding site detection covers the different applications above, the majority relates to small molecule pocket identification as a testimony of efforts to structure-based drug design of small chemical entities in recent decades. The accessibility to binding site identification is possible via standalone tools [16], websites [41], or databases of precomputed sites [35,42].

**Figure 1:**
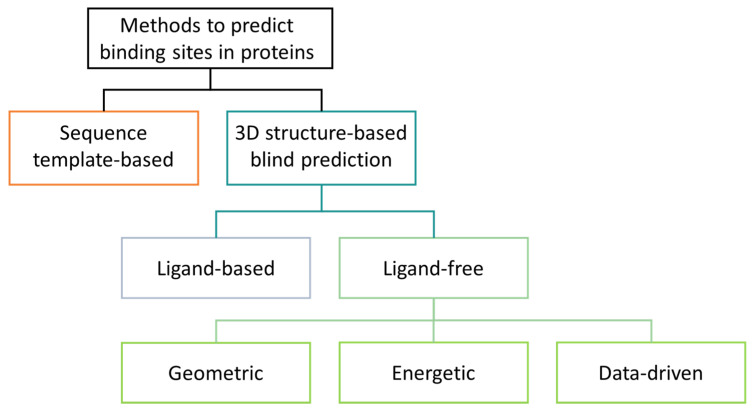
Classification of binding site detection methods.

The methods can be classified into three levels: (i) the genomic or 3D structure of the input, (ii) the dependency to bound ligands, and (iii) the class of the algorithm (Figure 1). Template or sequence-based methods such as ConSeq [43] identifies functionally important residues in protein sequences by searching for evolutionary relations with other proteins. Another approach is 3DLigandSite, which takes a protein sequence as input, although it relies on homology models or de novo structure predictions [41]. Structure-based pocket identification uses only the 3D coordinates of the structures as input and benefits from the augmentation of structural data [7].

Ligand-centric methods are restricted to protein–ligand complexes and is a site delimitation rather than a prediction. Noticeably, the analysis of crystallization additives binding sites might suggest potential allosteric pockets [44,45]. Typically, a site is defined as all residues within a certain distance cutoff to the partner’s heavy atoms, ca. 6 Å for protein–small molecule complexes. Alternatively, the set of residues can be restricted to those properly oriented toward the ligand, with the particularity that the distance cutoff varies according to the interaction type. These approaches are available through integrated environments, making it possible to manipulate protein structure coordinates and interactions such as Molecular Operating Environment (Chemical Computing Group, Montreal, Canada), or through independent tools for parsing protein 3D structure data [46].

Ligand-free approaches can operate on a larger range of structures, enabling the discovery of unprecedented sites. According to their search algorithm, they can be classified as geometric, energetic, or data-driven (Table 1). At first glance, all geometric methods aim at identifying sufficiently buried zones unoccupied by protein atoms, but they differ in their strategies to search for these areas. Grid-based methods place the protein into a cartesian grid and identify grid cells likely to be in a cleft by analyzing their neighborhood [13,14,47,48,49,50,51,52,53,54,55,56,57,58]. POCKET [13] and LIGSITE [12], two of the earliest methods, keep cells that correspond to a ‘protein-solvent-protein’ event by scanning in three and seven directions, respectively. VolSite annotates cavity points by pharmacophoric properties, complementary to that of the protein microenvironment [47]. Such algorithms are sensitive to grid resolution and orientation but are powerful to detect cavities of different sizes and curvatures. 

Contrarily, other methods process the protein coordinates directly and are not affected by the grid initialization phenomena. Based on the alpha-shape concept introduced by Edelsbrunner et al. [93], they circumvent protein cavities by connecting adequate adjacent Delaunay triangles via the ‘discrete flow’ method [6,59,60,69], or by clustering alpha spheres to satisfy pocket descriptors [16]. Alternative purely geometric approaches fill or coat the protein with spherical probes to delimit cavity void [61,62,63,64,65,66,67,94,95]. Finally, other concepts, such as monitoring the direction of surface normal vectors, were implemented [68].

The second category of ligand-free methods estimate favorable surfaces for protein–ligand contacts by calculating the potential energy of probes at different positions [78]. Generally, the Lennard-Jones potentials are used with hydrophobic probes. The nature and number of probes vary from a simple carbon probe in DrugSite [71] to 16 different ones in FTSite [72]. The potentials are either mapped to grid positions [70,71,72,73,74,75,76,77,78] or to probe the protein surface [79,80]. Evidently, the outputs of energy-based methods are influenced by the chosen force field. 

The final class of methods uses supervised models, trained on the features of well-characterized ligand binding sites. Hence, they differ in the features’ representation, training models, set of parameters, and datasets. P2RANK [84] is one of the examples based on classical machine learning models. The protein solvent-exposed atoms are processed into a topological and physicochemical feature vector which serves as input to a random forest classifier. Recently, many deep learning methods [86,87,88,89,90,91,92], majorly based on 3D-convolutional neural networks (CNNs) were introduced. CNNs have shown to be very powerful in image recognition problems [96] and were thus directly applied to protein binding sites represented as voxels with atomic attributes, while keeping the architecture of the CNNs previously used for other purposes. Another possibility to represent binding sites is used in PointSite [86], which addresses point clouds segmentation using sparse convolution. While these methods need to be challenged by prospective usages, recent advances in 3D point cloud deep learning [97] offer some wide perspectives for this type of problem.

Altogether, these methods have been evaluated on their performance to accurately predict binding pockets by comparing predictions on unbound proteins to ground truth ligand locations in their corresponding bound structures. Not only is the accuracy of the location analyzed, but also the delimitation or overlap with respect to the ligand [14]. The detected pockets might be too large or too small where a clustering is required. Thus, post-processing data generated by various tools may be useful [98]. Cleverly, meta-methods thrive to find consensus from different algorithms to increase the chances of correct predictions [99,100]. However, consensus might not always yield the right solution.

Indeed, all identified clefts do not necessarily correspond to the ability to accommodate a drug-like ligand (druggability). The concept of structural druggability [101] arose from observing the characteristics of pockets bound to pharmacological ligands: an average volume between 200 and 800 Å^3^, a good balance of hydrophobic and polar atoms enabling some binding specificity, and sufficient buriedness. A few methods were developed to predict target druggability [14,47,102,103,104,105,106]. Consistently, the topological and physicochemical characteristics of the pocket sites are encoded into descriptors and trained on curated datasets to generate classification models (e.g., support vector machines, random forest, linear regression). Since pocket druggability does not guarantee that the bound ligand will also be druggable, the term may be replaced by ligand-ability [107] or bind-ability [104]. For more information, we refer the reader to a recent review [108]. Interestingly, some of the previously described methods have implemented both a pocket detection and a rule-based druggability prediction [14,16,47,76], thereby enabling a straightforward selection of the most interesting pockets, notably for supramolecular assemblies [40]. 

## 3. Comparing Pockets: A Multi-Step Procedure

The methods that compare protein cavities operate in three steps: (i) describing the cavity with a suitable representation, (ii) comparing these representations, (iii) scoring the proposed comparison. Hence, successful results reside in a coordinated performance of each of these tasks. Yet, cavity representation, which is the first step of the procedure, is crucial as it influences the later steps. Generally, a poor representation in which relevant characteristics are missing cannot be compensated by the most efficient comparison or scoring algorithm. State-of-the art methods to compare protein cavities are summarized in Table 2. In the following sections, we will discuss the different approaches to achieve this end.

### 3.1. Pocket Representation

Once the pockets are delimited, the features are selected by considering different aspects. This step aims at focusing on the relevant characteristics that explain ligand recognition, while discarding unnecessary information. Our brain performs the same exercise on everyday life’s objects, for example, if we are asked to compare two cars, we might decompose the information into major aspects such as the brand, design, color, motor, etc. Interestingly, different people will focus on different combinations of these aspects, resulting in different decision making. For pocket modeling, there is the general knowledge that the attributes (size, physicochemical properties, flexibility) of residues flanking the site and their relative 3D location explain the specific recognition of ligands [17,27,132]. Therefore, pocket comparison methods approximate these residues into various representations which differ at three levels: (i) the discretization of the residues, (ii) the viewpoint, and (iii) the chemical features.

**Figure 2:**
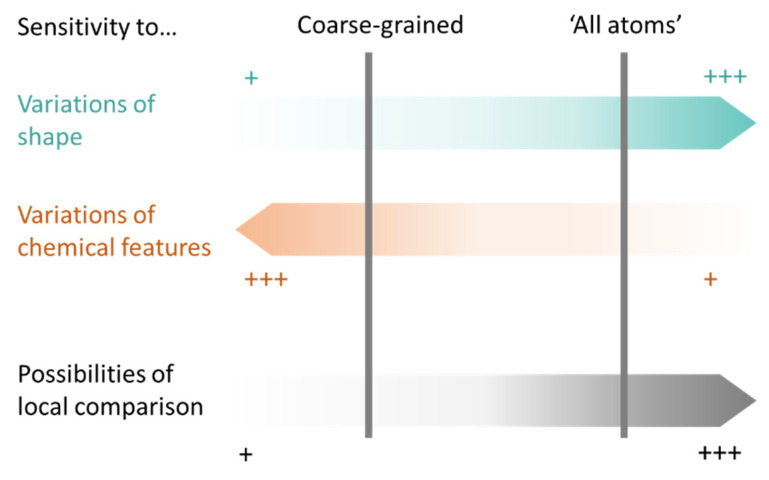
Sensitivity of coarse-grained to all-atom cavity representations to variations in atomic coordinates, chemical features, and subsequent applications (+: low, +++: high).

First, possible representations (Table 3), from coarse-grained to more detailed, can be a representative atom (typically the Cα or Cβ atom) describing an entire residue (e.g., Apoc), a group of pseudocenters or vectors associated with residue fragments (e.g., CavBase), a cloud of atoms (e.g., VolSite), or 3D voxels (e.g., DeepSite). The resolution of the representation determines how local the subsequent comparison can be. For example, the rigid matching of atoms which are 7 Å apart in a query pocket can only be associated with similarly spaced atoms in the reference pocket, therefore excluding a pertinent association of smaller areas. Resolution also influences sensitivity to chemical and coordinate variations (Figure 2). Coarse-grained representations are less sensitive to variations in atomic coordinates but are more perceptive of changes in chemical properties such as single residue mutations. They offer a better signal-to-noise ratio at the cost of information. In grid-based approaches, the grid resolution (often 0.5 to 1.5 Å) is adjusted to capture the shape of the site, while compromising between precision and computing [47,124]. Although small changes of residues are reflected in detailed representations, they can be perceived to a lesser extent since drowned in many other information elements. The detection of such details is highly influenced by the assignment of chemical features and the performance of the search algorithm. Noticeably, some methods have adopted a mix representation scheme, wherein gross representations are used for a faster search and finer representations are involved in the scoring [17]. 

**Figure 3:**
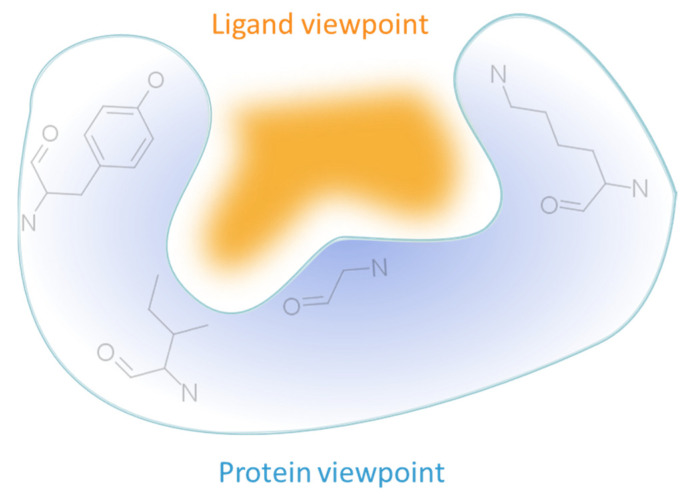
Protein cavity representation according to the protein or the ligand perspective.

Secondly, most methods adopt the protein perspective by considering atoms or pseudocenters at the protein surface (e.g., FuzCav, SMAP). A few stand out by projecting these protein patterns into the ligand space, wherein polyhedron, voxels, or points are annotated with the properties of nearest or well-oriented protein features (e.g., IsoMIF, SiteAlign) (Table 3). Such discretization aims at offering a good balance between information completeness while handling variations in atomic coordinates and features. However, it is important to recall that grid-based representations are affected by the centroid location and axes orientation during the grid initialization. As a result, the distribution of feature types might change (a protein feature might move in adjacent voxels or not be represented at all), particularly when a voxel is associated with only one feature at a time. The same representation (e.g., cloud of points) can be applied to either key protein atoms [116] or grid points delimiting the accessible cavity space [129], thereby offering the possibility to mirror an imaginary ligand viewpoint and providing an alternative comparison approach (Figure 3).

Finally, besides the two aspects described above, the methods differ in their definition of chemical and geometric features. For example, Binkowski et al. do not consider the chemical type of atoms but showed that the relative position of the surface atoms describing the shape of the pocket already contains some discriminative information [113]. However, shape information alone is insufficient; hence, it is not surprising that almost all the state-of-the-art site comparison methods annotate surface coordinate atoms with pharmacophoric features to improve the discrimination between redundant areas. In coarse-grained representations, Cα/Cβ atoms are annotated according to the chemical groups of their residues. For instance, APoc defined eight exclusive chemical groups, allowing for a residue to belong to only one [28]. Searching for the identity of chemical features between the query and reference pockets with such representations does not account for the interchanging role that fragments in different amino acids may have. For example, the hydroxyl group of serine and tyrosine can be a hydrogen bond donor or acceptor, as tyrosine additionally displays an aromatic feature; yet serine and tyrosine belong to different classes. To correct this effect, residues can be assigned multiple classes (e.g., SiteAlign). Alternatively, single or groups of atoms defining pseudocenters are annotated according to their interaction capacities (e.g., a histidine side chain is represented by a hydrogen-bond donor–acceptor feature and aromatic pseudocenters in CavBase). Commonly, five to eight pharmacophoric features are defined (KRIPO, SiteEngine, VolSite), as well as up to more than 40 atom types (e.g., PocketFEATURE). Other possible chemical attributes are partial charges used in P.A.R.I.S (sup-CK) or SiteEngine scoring, atomic density (SuMo), or atom types (e.g., SitesBase). The definition of many feature types might improve the description of the site with precision but might at the same time hinder remote similarity detection by narrowing the applicability domain of the method. Aside from the chemical features, the geometrical patterns are sometimes considered: CavBase and RAPMAD indicate the directionality of polar features by vectors, SuMo considers the directionality of the patterns toward the cavity by scalar triple product, SOIPPA assigns normal vectors to local surfaces, TrixP and Sitelign consider distances to fixed points.

In a nutshell, there are various ways to represent a protein cavity. Challenges reside in finding a good balance between comprehensive representation of features to ensure reliability and loose representation making it possible to detect remote similarities. While the absence of pocket attributes cannot be recovered at the later comparison step, too many attributes may constitute difficulties for the search algorithm in separating the signal from the noise.

### 3.2. Similarity Search

**Figure 4:**
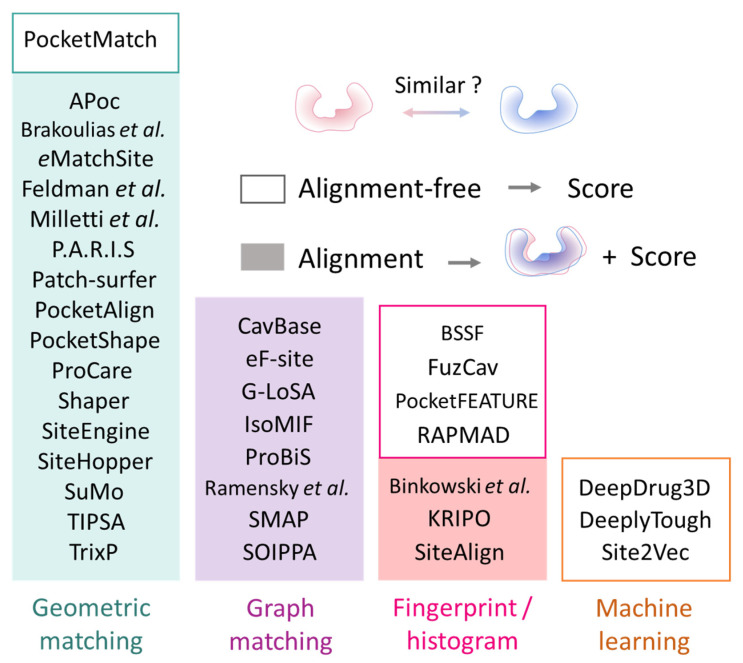
Classification of state-of-the-art methods for protein pockets comparison. Alignment-based methods compute a transformation (rotation, translation) to superpose the query to the target site.

Following the selection of features characterizing the cavities, similarity is estimated by algorithms that search for common patterns shared between two sites. First, the representations of the protein cavities are converted or organized into comparable and computer-friendly objects that can be processed automatically. There are a variety of search algorithms to this end, which can be categorized according to their inputs, procedure, and visual interpretability (Figure 4).

The first category of algorithms searches for geometric (e.g., pairwise distances, angles, shape) and chemical (identical or compatible types) constraints to match. It is not safe to expect a perfect match, given the errors in 3D structure resolution, the flexibility nature of proteins, and the aim to find unobvious similarities. Therefore, a certain margin of geometric errors is always tolerated. PocketMatch compares set of distances belonging to 90 combinations of atom types and properties to establish correspondences between two pockets and keeps the solution maximizing the number of correspondences [19]. Global alignment methods (P.A.R.I.S, SiteHopper, Shaper) try to maximize the overlap between two cavities. A seed alignment is initialized, for example, by superposing the centroids or principal axes of the two sites, which are then optimized [47,116,133]. SiteHopper and Shaper rely on the OpenEye’s ROCS (OpenEye Scientific Software, Santa Fe, NM, USA), wherein atoms/points are represented by smooth Gaussians to enable fuzzy shape comparisons [47,127]. A different approach for global optimization is to establish seed correspondences. APoc compares local protein fragments [28], Milletti et al. associate points based on their circular fingerprint similarity [115], eMatchSite assigns seven residue-level scores at selected Cα atoms [29], Patch-Surfer compares the patch surface properties by 3D functions [119]. The next alignment is solved by the Hungarian algorithm or other combinatorial optimization algorithms [8,22,102,115]. PocketAlign uses a similar approach using BLOSSUM62 weights when generating local seed alignments, which are later extended to the full structures [117]. Alternatively, some methods partition the pocket by considering a few points each time. Given that at least three points are necessary to superpose two objects without ambiguity, those methods enumerate triplets or quadruplets of feature points in the query to iteratively search for equivalent cliques in the target [17,30,110,111,120,121,129]. The formation of the n-tuples can be customized to avoid promiscuous sets. In TrixP, triangles solely made of hydrophobic features are not considered [121]. A match can signify a simple correspondence of identical chemical types and pairwise distances (SiteEngine, TIPSA) or of additional properties such as vector angles, local shape (ProCare, TrixP). ProCare relies on a 41-bin histogram describing each point, accounting for both shape and pharmacophoric features [129]. The alignment is performed in two steps, first by finding equivalent pocket points using a random sample consensus algorithm [134], then iteratively refining the preliminary alignment by the iterative closest point (ICP) method [135]. Aligning all possible combinations is costly in time, hence SiteEngine and TrixP, employ hashing and bitmap indexing, respectively, allowing for a ‘search IN’ for the faster identification of similar patterns.

In the second category, selected points form the nodes of a graph. According to the cavity representation, each node is annotated by a property and the edges by their lengths. Comparing two cavities results in comparing two graphs to extract the maximum common subgraphs. To achieve this end, a product graph is built by associating similar nodes (property comparison) and edges of almost equal distances, tolerating a certain deviation. Cliques are identified in this association graph to derive pairs of equivalent points that can be used to superpose the two cavities. CavBase, G-LoSA, ProBiS, etc. (Figure 4) are based on this principle. Differences between methods arise from the graph construction (minimal and maximal distances to consider adjacent nodes), distance tolerances, and the definition of a property match (identity or compatibility). For example, G-LoSA explores three different distance deviations (1.5, 2.0 and 2.5 Å) and further evaluates the alignment of local triangles within each clique of at least four nodes [126]. Clique detection is computationally expensive, particularly with dense graphs (e.g., 0.5 Å grid, [124]). Therefore, it requires practically efficient solutions such as the Bron–Kerbosch algorithm [136] and improved variants [137].

Methods in the third category generally adopt a global vision of the protein cavity. They consider a pocket as a fixed-length fingerprint or histogram, and comparing two pockets is amounts to calculating the similarity or distances between their fingerprints/histograms. BSSF, FuzCav, and KRIPO compute couples or triplets of pharmacophoric features separated by binned distances [21,24,25]. While the two former count the number of occurrences of each combination, bits are activated in KRIPO when a combination occurs. Then, KRIPO fuzzifies its fingerprints to account for the neighborhood phenomena [21]. SiteAlign also compares fingerprints but, contrarily to the other methods, the fingerprint of the query pocket is iteratively generated, as it derives from properties of the cavity projected on a rotated/translated 80-face polyhedron [20]. Since the binding site is discretized and a finite number of geometric transformations are sampled, the performance of the search depends on the resolution of the steps, at the cost of computing time. Finally, Binkowski et al. [113] and RAPMAD [26] compare the distributions of pairwise distances between the pocket features. RAPMAD generates 14 histograms, one for each of the seven pharmacophoric features, considering two centroids. The idea behind these implementations is that similar binding sites exhibit similar sets of distances. However, these methods may suffer from matching redundant distances that do not superpose geometrically. The advantage of fingerprints/histograms is to enable a faster comparison, without the computationally expensive alignment. Still, KRIPO and Binkowski et al. generate an alignment independently of the comparison procedure for visual inspections, with SiteAlign as part of its search procedure.

Finally, the recent regain of interest for deep neural networks on chemical information favored the emergence of data-driven methods for binding site comparison. Typically, binary classification models are created to discriminate between similar and dissimilar pairs of pockets. Site2Vec transforms the features representing a cavity into a fixed-length vector that can feed a random forest classifier [131]. DeepSite, DeepDrug3D, and DeeplyTough discretize the 3D space of the pocket as voxels, and logically train a convolutional neural network (CNN) binary classification model [32,92,128]. Besides the dependency on sufficiently diverse training datasets for a generalized model, these approaches suffer from the interpretability of the predictions. Interestingly, DeepDrug3D exploits the activation map to highlight areas that largely contribute to the classification.

All the above summarized methods use the protein information only for comparison. Provided that a pocket is delimited, they have a larger scope that may reach target deorphanization [36]. When bound ligands are available, comparing the protein–ligand interactions can be an efficient alternative, particularly when the goal is to reproduce existing binding modes. Likewise, dedicated methods aimed at aligning interaction patterns are based on graph alignment or fingerprint matching [138].

### 3.3. Local Comparison of Protein Cavities

**Figure 5:**
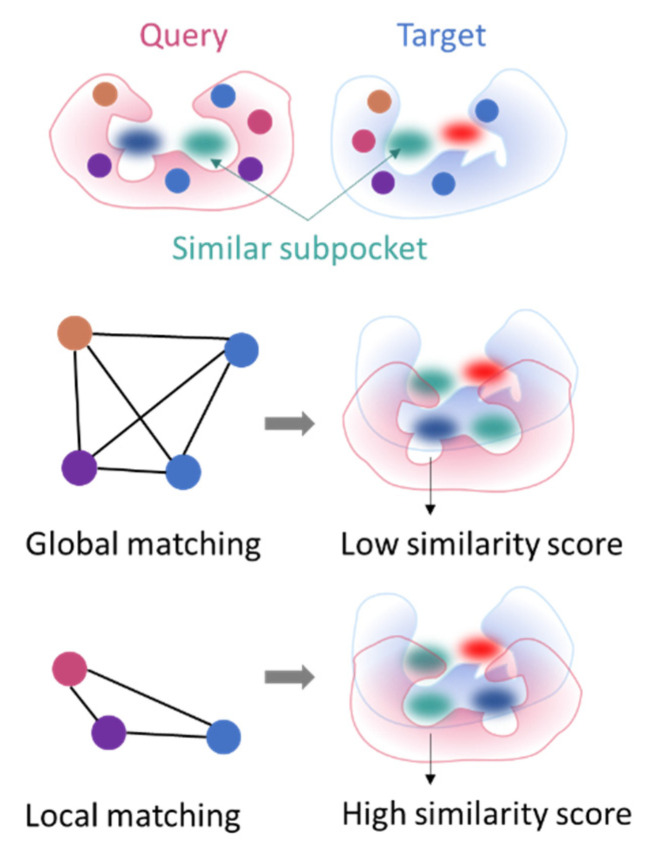
Global versus local pattern comparison.

Looking for an average match that maximizes the overlap between entire cavities is not necessarily the right solution to similarity estimation. Local comparison is a popular term, often used to differentiate full protein structural comparison from protein site comparison. Here, we refer to the truly local comparison of protein pockets (Figure 5), i.e., subpockets of approximately 3 to 4 Å radius (for reference, approximately the shortest distance between a chain of four atoms connected by simple bonds). 

Enabling local similarity detection is relevant for drug design applications since a few similar subpockets between two targets may suffice for a same ligand to bind. This observation was applied to explain the binding of cyclooxygenase type 2 inhibitors to carbonic anhydrase [22]. Local comparison is notably suitable to handle cases of protein/ligand conformational change upon ligand binding [115]. 

Logically, methods that can operate locally have implemented detailed site representation and/or adequate algorithms that partition the cavity during the search. In G-LoSA, global matches are decomposed into local subsites to generate other solutions [139]. Local comparison can also be achieved by providing subpockets as input into the search algorithm. KRIPO [21] and ProCare [129] make it possible to compare subpockets delimited by fragmented ligands. While the search algorithms are crucial for identifying zones of similar patterns in two pockets, how these similarities are quantified is equally important, since generalizing the scoring over the full pockets might hinder any local similarity as well. By analogy with ligand versus fragment promiscuity, comparing smaller cavity regions is likely to be more redundant at the proteome scale than comparing full cavities, enabling to catch similarities between remote proteins but at the same time yielding possible unspecific matches that need to be discarded by robust scoring functions to quantify pocket similarity. 

### 3.4. Scoring Functions

Scoring functions serve two purposes: (i) guiding the alignment by discarding unrealistic solutions and prioritizing the best matches, (ii) quantifying the estimated similarity between the pair of pockets to consider. It is not uncommon to use distinct scoring functions for the alignment search and its final quantification [18]. Consequently, a method may implement an accurate representation and efficient search algorithm but may fail to accurately predict similarity levels if the scoring function is incorrectly calibrated. Some analogy can be made with the problem of pose sampling and ranking in docking, leading to rescoring efforts. 

Aspects to consider when defining a scoring function for binding site comparison are (i) the discriminative potential, (ii) the minimal and maximal boundaries, (iii) the broadness, (iv) the sensitivity to the size of the cavities, and (v) the interpretability. The very simple and intuitive scoring scheme counts the number of common patterns between two pockets (Brakoulias et al.) [111]. However, bigger pockets may tend to score higher as the chances for a match increase. To avoid this bias, methods account for the size of the pockets using metrics such as the proportion of aligned features with respect to the query/target size (FuzCav, PocketMatch), Tanimoto indices (IsoMIF, KRIPO, TIPSA, Shaper), and Tversky indices (ProCare, Shaper). SiteHopper adopts a linear combination of Tanimoto measures for shape and chemical features matching. Almost all alignment-based geometric matching methods aim at minimizing the root mean square deviation (RMSD) of superposition candidates or with respect to a cutoff (Brakoulias et al., SuMo, etc.). In some cases, the RMSD is also a composite of the final score (Milletti et al., PocketAlign). In the same way, the CavBase R2 score accounts for the RMSD of pseudocenters when scoring the overlap of the surface grid points. Implementing successive scores (Binkowski et al., ProBiS) allows the user to apply a custom filter according to the desired application or suggests a hierarchical scoring. For instance, SiteEngine proposes a workflow wherein a gross evaluation makes it possible to rapidly filter out bad solutions before applying a finer rescoring on promising matches. Instead of reporting similarities, some methods measure the distances between pockets (SiteAlign) instead—the lower, the better. BSSF and RAPMAD, which compare histograms, respectively report the Kolmogorov–Smirnov and the Jensen-Shannon divergences. Scoring functions can be more complex, often at the cost of interpretability (Feldman et al., eMatchSite, P.A.R.I.S).

Weights are used to give more or less importance to different variables (types of features, geometric patterns) but their assignment is at best subjective [119,121,139], intuitive such as inverse of feature frequency, or adapted from sequence alignment methods (BLOSSUM, PSSM) [114,117,140]. Proportioning penalties of mismatches with respect to the positive contributions of the matches (e.g., TrixP) is tricky and may ameliorate or worsen the discrimination performance in context-dependent noisy representations. In fingerprint comparisons, bins are populated with counts or integer descriptors with variable ranges. The descriptors are normalized [20] or the scores are corrected to account for the increase in activated bits with respect to the size of the cavity [21]. Finally, the commutativity of the score should be regarded to ensure a consistent output whatever the reference/query order.

A few studies [30,47,129,141] have assessed the significance (Z-score, *p*-values) of the scoring function by analyzing random distributions or robustness to variations in the cavities (simulated data, molecular dynamic simulations). While these studies offer a certain overview on possible scoring thresholds in screening settings, we draw attention to potential bias in setting up calibration datasets.

## 4. Retrospective Evaluations and Datasets

To demonstrate their applicability, the methods for comparing protein binding sites have been evaluated for their ability to (i) discriminate between similar and dissimilar binding sites (classification), (ii) retrieve similar pairs seeded in decoys (enrichment), and (iii) cluster proteins belonging to the same families according to other classifications (e.g., SCOP, functional annotations). The availability of structural data impacts the design of the evaluation datasets.

As for any benchmarking study, the quality of the dataset is instrumental to the reliability of the conclusions. Ligand-based and structure-based virtual screening benefit from well-established standards and datasets [142,143]. Predicting the binding affinity of molecules to a target can be directly verified by experimental measures in many circumstances. Contrarily, pocket similarity cannot be measured experimentally. Instead, similarity prediction suggests hypotheses such as the recognition of similar ligands or the catalysis of the same reaction, which are then confronted with in vitro experiments. What is conveyed here is that there is not a straight line between predictions and verifications since ligand recognition involves other parameters likely not evaluated by binding site comparison methods, such as the pocket flexibility, the influence of disregarded parts of the protein, and the ligand conformations and energetics. Indeed, the ligand may bind to different proteins in different conformations and use different interaction patterns [144]. 

Nevertheless, many available datasets [24,28,116,128,144,145,146] have been set-up with the assumption that similar pockets bind to identical or similar ligands, and vice versa (Table 4). These include proteins belonging to the same family for the easiest ones, and unrelated proteins for the most difficult datasets. In these cases, unrelated proteins are predicted by other computational approaches (sequence alignment, global structural comparison). Besides the discussions above, one issue encountered with these definitions is how to set the similarity cutoff to cluster binding sites and ligands.

Chen et al. defines similar pairs as pockets in proteins sharing at least three submicromolar ligands, while dissimilar pairs share at least three ligands large affinity variations going from one target to the other [133]. However, from a medicinal chemistry perspective, this dataset is imbalanced as the number of similar pairs largely exceeds that of dissimilar pairs (Table 4). Still, a main concern is that structural data evidencing that the proposed pair of binding sites effectively accommodating the same ligand are usually missing. Generally, datasets relying on ligand binding information suffer from data incompleteness [147]. Dissimilar pairs are based on limited available/accessible binding information, while all ligands have not been tested against all targets. Otherwise, pairs labeled as ‘dissimilar’ might have fallen into the ‘similar’ classes.

**Table 4:** Common datasets used in benchmarking studies for pocket comparisons.

Purpose	Name	Content	# Positive (# Negatives)
Pairs of cavities from dissimilar proteins binding identical or similar ligands (positives) and dissimilar ligands (negatives)	APoc set [28]	Diverse	38,066 (38,066)
	Barelier et al. [144]	Diverse	62
	Homogeneous [116]	Diverse	100
	Kahraman [146]/extended [116]	Cofactor sites	100/972
	sc-PDB subset [47]	Diverse	1070
	TOUGH-M1 [145]	Diverse	505,116 (556,810)
	TOUGH-C1 [128]	Nucleotides, heme, steroid sites	2218
Pairs of proteins sharing 3 high affinity ligands (potency < 100 nM) vs. pairs of proteins sharing 3 ligands with divergent affinities	Vertex [133]	Diverse	6598 (379)
	Vertex refined [129]	Diverse	338 (338)
Pairs of cavities of associated with the same (positives) or different (negatives) functions and fold class	sc-PDB subset [24]	Diverse	769 (769)
	sc-PDB subset [121]	Diverse	766 (766)
	sc-PDB subset [129]	Diverse	383 (383)
Intra-family classification	Proteases, kinases, GPCRs, Estrogen receptors [17,20,47,115,148]		-
Difficult cases	Difficult cases [19,24]	Diverse from experimental validations	8
Successful applications	ProSPECCTs D7 [38]	Diverse from experimental validations	115 (56,284)
Structures of identical sequences	ProSPECCTs D1 [38]	Diverse	13,430 (92,846)
	ProSPECCTs D1.2 [38]	Diverse	241 (1784)
NMR structures	ProSPECCTs D2 [38]	Diverse	7729 (100,512)
Artificial sets: random mutations	ProSPECCTs D3 and D4 [38]	Diverse	13,430 (67,150)

Given the bias in the PDB dataset towards some protein–cofactors complexes and well-studied protein families, methods have been extensively evaluated on nucleotide-binding pockets [146], although such test cases are quite specific or far too easy to be really predictive of real drug discovery scenarios. Similarly, the capacity of binding site comparison tools to cluster together binding sites originating from the same protein family (e.g., proteases, kinases, or steroid-binding sites) have been widely studied [17,20,115,148]. Alternatively, other datasets proposed pairs of similar and dissimilar sites based on functional annotations [149] or folds [150,151]. Starting from really druggable protein–ligand complexes [152] is often advised in the case of medicinal chemistry applications [20,24,129,138]. Due to the increasing accuracy of deep learning methods [153,154] to predict protein structures with near-atomic resolution, the druggable pocketome is predicted to significantly expand in the next years [155]. Therefore, clear guidelines, as those recently proposed in ProSPECCTs [38], are welcome. Many artificially built datasets are too easy or do not correspond to realistic challenges. Compilations of difficult cases drawn from experimental observations are provided, but such examples are rare [19,24,121]. 

## 5. Prospective Applications

The best possible validation method of any binding site comparison tool is indeed to experiment. True prospective validations (Table 5) are still rare for several reasons:

- Fragment/ligand promiscuity towards unrelated targets of known 3D structure remains are a rare event [156];
- Direct drug repurposing from in silico [157] or in vitro screening strategies have not yet yielded any success in terms of new indication approvals [158], as recently exemplified by the COVID-19 pandemic;
- The experimental validation of putative binding site similarities is not as straightforward as testing many compounds on a single target. For every putative off-target, a suitable assay has to be used if available, or more likely needs to be developed on purpose. In vitro biophysical assays (e.g., NMR, thermal shift) give a direct answer of shared ligand binding to two different targets [159,160] but do not necessarily evidence the binding site location, by opposition to enzymatic assays [40,161,162,163,164] or binding competitions experiments for which the binding site is usually unambiguous [165,166,167]. If not possible otherwise, functional and/or in vivo assays [168,169] can be used but are more difficult to interpret since the examined function might be biased by binding to another target.
  Known success stories (Table 5) have notably enabled:
- The explanation of target-mediated side effects and guidelines to optimize the ligand selectivity by suitable structural modifications [168,169];
- The explanation of off-target beneficial effects [165];
- The validation of cross-docking data for repurposing hypotheses [162,166];
- The confirmation of ligand 2D and 3D shape similarities [164];
- The serendipitous discovery of remote similarities across totally unrelated targets during code benchmarking and validation [159,167].

**Table 5:** Examples of small molecular weight ligand-binding site comparisons relevant to medicinal chemistry.

Method	On-Target	Secondary Target	Ligand	Secondary Target Affinity	Ref.
SOIPPA	Estrogen receptor alpha	SERCA Ca^2+^ ion channel ATPase	Tamoxifen	IC_50_ = 5 µM	[168]
CPASS	Bcl-2 apoptosis protein Bcl-xL	Type III SS Needle Protein (PrgI)	Chelerythrine	N/A ^a^	[160]
SOIPPA	Catechol-O-methyltransferase	Enoyl-acyl carrier protein reductase	Entacapone	IC_50_ = 80 µM	[162]
SiteAlign	Pim-1 kinase	Synapsin I	Quercetagetin	IC_50_ = 0.15 µM	[167]
SMAP	HIV-1 protease	ErbB2 receptor tyrosine kinase	Nelfinavir	N/A ^b^	[163]
PSSC	Monoamine oxidase	Lysine-specific demethylase 1	Namoline	IC_50_ = 51 µM	[166]
SMAP	Epidermal growth factor	β-secretase	Gefitinib	IC_50_ = 20 µM	[165]
KRIPO	Cannabinoid type 1 receptor	Adenine nucleotide translocase 1	Ibipinabant	N/A ^c^	[169]
PSIM	PPAR gamma	Cyclooxygenase type 1	Fenofibrate	IC_50_ = 950 μM	[161]
TM-align	Receptor Tyrosine kinases	Acetylcholinesterase	Pazopanib Sunitinib	IC_50_ = 0.93 μM IC_50_ = 5.87 μM	[164]
Shaper	Cyclooxygenase type 1	Cinnamoylesterase	Flurbiprofen	IC_50_ = 400 µM	[40]
ProCare	HIV-1 reverse transcriptase	TNF-α trimer	Efavirenz Delavirdine	Kd = 24 µM Kd = 49 µM	[159]

^a^ binding evidenced by ^15^N-^1^H NMR-HSQC spectra; ^b^ 15% inhibition at 20 µM in a kinase activity assay; ^c^ 30% Inhibition of ANT-dependent mitochondrial ADP uptake at a concentration of 100 µM.

The above-cited examples share common characteristics. First, the repurposed ligands usually exhibit (very) weak affinities towards the secondary target, notably when the on- and off-targets are unrelated. In all cases, the studied ligand needs to be optimized for potency and selectivity towards the secondary target, thereby abolishing the benefits of immediate in silico-guided drug repurposing [159,160,161,162,163,164,165,166,167,169]. Second, and in relation to the first observation, the noticed pocket similarity is usually local and not global. In other words, only the subpockets of the two targets under investigation account for the shared ligand binding. This explains why some targets, notably those exhibiting hydrophobic subpockets (COX-1, HIV-1 RT, PPARγ, ER-α) are frequently observed among the protein pairs cited below (Table 5). The conservation of shared polar and apolar pocket features is a rarer event but leads to higher affinities of the corresponding complexes [164,167]. 

## 6. Conclusions

This review presents the current state of ligand-binding site comparison applied to small molecule drug design. As computer-aided drug design strategies, assessing the similarity of protein pockets constitutes a unique way to analyze structural information, as they complement other well-spread approaches. The repertoire of available methods is diverse with respect to the detection and representation of cavities, the search algorithms, and the scoring functions. All of these aspects must somehow be coordinated to achieve the best performance. Still, the limitation of experimental data and biases in datasets represent major obstacles to properly evaluate such methods. In reality, estimating protein site similarity is always context-dependent. The importance of matched features is influenced by the chemical context and physicochemical considerations of the targets, making it hard to predict subtle and specific similarities from generalized principles. 

One holy grail of computational chemists is to repurpose existing drugs proposed by structure-based experiments. Although this pursuit appears at best hardly probable due to the optimization of drugs for their on-targets [157,158], we believe that binding site comparisons are the most useful in finding not global but local similarities, and therefore to repurpose fragments [22] and not full ligands, provided that the selected fragments can be grown or linked to enumerate full ligands or target-focused libraries [170].

Binding sites comparisons have demonstrated an effective contribution to medicinal chemistry projects, from the elucidation of previous biological observations to the generation of new hypotheses supported by experimental validation. The majority of the state-of-the-art methods are based on the superposition of the compared structures. The alignment allows for a visual inspection and increases the possibilities of applications. Typically, pocket-bound ligands in the reference frame can be transposed into the target pocket and serve as a starting point for ligand generation. The improvement of the algorithmic efficiency of the methods alongside with technological progress may enable to better follow the current growth of publicly available protein structures, determined experimentally or predicted at near-atomic resolution [171]. 

## Figures and Tables

**Table 1:** Common structure-based methods to predict ligand binding pocket in proteins.

Category	Search Approach	Methods
Geometric	Grid	CAVER [51], CAVIAR [49], DoGSite [14], ghecom [57], KVFinder [48], LIGSITE [12], LIGSITEcsc [53], McVol [58], POCKET [13], PocketDepth [55], PocketPicker [54] VICE [56], VOIDOO [52], VOLSITE [47]
	Alpha-shape	APROPOS [59], CAST [6], CASTp [60], Fpocket [16],
	Spherical probes	DEPTH [61], HOLE [62], HOLLOW [63], PHECOM [57], PASS [64], Roll [65], SURFNET [66], SURFNET-ConSurf [67], Xie and Bourne [33]
	Other	MSPocket [68], SplitPocket [69]
Energetic	Grid	AutoLigand [70], DrugSite [71], FTSite [72], PocketFinder [73], Q-SiteFinder [74], SITEHOUND [75], SiteMap [76], pocket-finder [77], GRID [78]
	Spherical probes	dPredGB [79], Morita et al. [80]
	Other	Gaussian Network Model [81]
Data-driven	Machine learning	GRaSP [82], MCSVMBs [83], P2Rank [15], PRANK [84], SCREEN [85]
	Deep learning	PoinSite [86], DeepPocket [87], PUResNet [88], DeepSurf [89], BiteNet [90], Jiang et al. [91], DeepSite [92], ISMBLab-LIG [91]

**Table 2:** Methods to compare protein cavities.

Year	Name	Detection	Principle	Scoring	Evaluation Datasets
2002	CavBase [17]	LIGSITE [12]	Clique detection in graphs of pseudoatoms	Overlap of surface grid points, RMSD	Cofactor sites, kinases, serine proteases
2002	eF-site [109]	Ligand Databases	Clique detection in graph of surface normal vectors and electrostatic potentials	Normalized and weighed contributions of vectors angles, potentials, distances	Phosphate sites, antibodies, PROSITE classes
2003	SuMo [110]	Ligand	Incremental match of triplets of pseudocenters	Count of matches, RMSD, composite of Euclidean and density distances	Protease catalytic sites, lectines
2004	SiteEngine [18]	Ligand	Match of triplets of points by hashing	Hierarchical scoring: count of matches, RMSD, overlap of patches, local shape	Cofactors, steroids, fatty acid sites, catalytic triad in proteases
2004	SitesBase [111]	Ligand	Match of triplets of points	Count of matches, RMSD	Cofactors, phosphate sites
2007	Ramensky et al. [112]	Ligand	Clique detection in graph of atoms	Dice similarity of matches	Diverse
2008	Binkowski et al. [113]	CAST [6] Ligand	Comparison of pairwise distance histograms	Kolmogorov–Smirnov divergence, overlap of volume, RMSD	Cofactor sites, HIV proteases
2008	PocketMatch [19]	Ligand	Comparison of sorted pairwise distances	Normalized count of matches	Diverse, SCOP classes
2008	SiteAlign [20]	Ligand	Alignment of polyhedron fingerprints	Normalized distances of fingerprints	Proteases, kinases, estrogen receptors, GPCRs
2008	SOIPPA [114]	Ligand	Clique detection in graphs of atoms	Composite weighted by frequencies, PSSM, distances	Cofactor sites, SCOP classes
2009	SMAP [33]	Ligand	Clique detection in graphs of atoms	Gaussian densities from distances, angles of normal vectors, BLOSSUM weights	Cofactor sites
2010	BSSF [25]	PASS [64]	Comparison of fingerprints of binned distances and properties	Canberra distances of fingerprints	Diverse, synthetic data, SCOP classes
2010	Feldman et al. [30]	Ligand	Match of subsets of Cα atoms	Probabilistic score from distances between matches	Diverse, kinases
2010	FuzCav [24]	Ligand	Fingerprints of triplets of atom features	Maximal proportion of matches	Diverse, functional groups, 8 difficult cases
2010	Milletti et al. [115]	Ligand	Comparison of 3 concentric spheres fingerprints encoding neighborhood for each point, solving linear assignment	Composite of fingerprint distances and RMSD	ATP sites, kinases
2010	P.A.R.I.S (sup-CK) [116]	Ligand	Initial alignment optimized by gradient ascent to maximize a Gaussian kernel	Gaussian kernel	Cofactor sites
2010	ProBiS [31]	Ligand	Maximum clique detection in graphs of surface atoms	Count of Matches, RMSD, angle between vectors	Cofactor/metal sites, protein–protein interfaces, protein–DNA complexes
2011	PocketAlign [117]	Ligand	Initial pairs from sorted lists of atom distances, then extend	Count of matches, RMSD	Cofactor sites, SCOP classes
2011	PocketFEATURE [118]	Ligand	Comparison of 7 concentric spheres fingerprints encoding neighborhood for each microenvironment	Normalized Tanimoto similarity of fingerprints	Kinases
2012	KRIPO [21]	Ligand	Fingerprints of triplets of pharmacophore	Modified Tanimoto of fingerprints	Diverse, fragments subpockets, search of bioisosteric substructures
2012	Patch-Surfer [119]	Ligand LIGSITE [12]	Comparison of 3D Zernike of surface patches solving a weighted bipartite matching	Composite of surface match distances and size differences	Cofactor sites
2012	Shaper [47]	VolSite [47]	Comparison of cloud of points by Gaussian shapes matching	Tanimoto, Tversky of matches	Diverse, GPCRs, proteases
2012	TIPSA [120]	Ligand	Match of quadruplets of points, iterative refinement by Hungarian algorithm	Tanimoto of matches, overlap of volume, normalized RMSD	Cofactor sites
2013	Apoc [28]	Ligand CAVITATOR [28], LIGSITE [12]	Seed alignment by comparing secondary structures, optimized by solving linear assignment problem	Composite of vector orientation, distance, properties	Diverse, similar ligand recognition sites
2013	TrixP [121]	DoGSite [122]	Search for common shape and triplets of points by bitmap indexing	Composite of matches count, angle between vectors, mismatches penalty	Diverse, 8 difficult cases, protease, estrogen receptor, HIV reverse transcriptase
2014	*e*MatchSite [29]	*e*FindSite [123]	Template-based alignment optimized by Hungarian algorithm	Machine learning score: RMSD, residue, properties	Cofactors, steroid sites
2014	RAPMAD [26]	LIGSITE [12]	Comparison of 14 pairwise distance histograms, one for each property	Jensen–Shannon divergence of histograms	Cofactor sites, proteases, diverse
2015	IsoMIF [124]	GetCleft [125]	Clique detection in graphs of interaction grid points	Tanimoto of descriptors of matched points	Cofactors, steroid sites
2016	G-LoSA [126]	Ligand	Clique detection in graphs of atoms	Feature-weighted count of matches	Diverse, Ca+ sites, similar ligands recognition sites, protein–protein interfaces
2016	SiteHopper [127]	Ligand	Comparison of surface atoms by Gaussian shapes matching	Weighted combination of shape and color Tanimoto	Diverse using binding affinities
2019	DeepDrug3D [128]	Ligand	Convolutional neural network model	Binary classification	Cofactors, steroids sites, proteases
2020	DeeplyTough [32]	Fpocket [16] Ligand	Convolutional neural network model	Binary classification	Cofactor sites, diverse and using binding affinities
2020	ProCare [129]	VolSite [47]	Match of randomly sampled quadruplets refined by iterative closest point	Tversky of matched pharmacophoric properties	Diverse, using functional annotation, fragments subpockets, search of bioisosteric structures
2021	PocketShape [130]	Ligand	Initial alignment optimized by Hungarian algorithm	Composite of matches, orientation of residues	Diverse SCOP classes, kinases
2021	Site2Vec [131]	Ligand	Random forest model on autoencoder-generated descriptors	Binary classification	Cofactors, steroid sites, diverse

**Table 3:** Discretization of the residues to represent a protein cavity.

Representation	Illustration ^a^	Methods
Single points	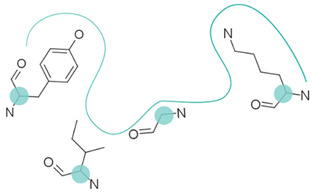	APoc, eMatchSite, FuzCav, G-LoSA, PocketAlign ^b^, SiteAlign ^b^, SMAP, SOIPPA,
Pseudocenters	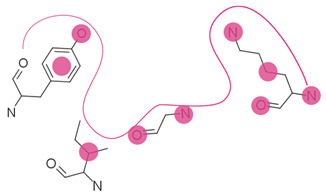	BSSF, CavBase ^b^, KRIPO, PocketAlign ^b^, PocketMatch, RAPMAD, Site2Vec, SiteEngine, SuMo, TrixP ^b^
Surface points, surface patches, volume points, polyhedron	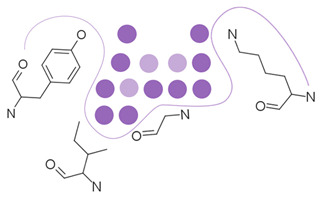	CavBase ^b^, DeepDrug3D, DeeplyTough, IsoMiF, Patch-Surfer, ProCare, Shaper, SiteAlign ^b^, TrixP ^b^
All heavy atoms	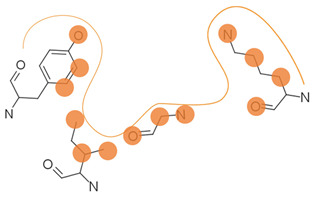	Binkowski et al., Brakoulias et al., Milletti et al., P.A.R.I.S, ProBiS, SiteHopper, TIPSA

^a^ The protein cavity is delimited by a few residues. Representative points at different resolutions are depicted as colored spheres. ^b^ Some methods use mixed representations.
